# Exposure to iodine, essential and non-essential trace element through seaweed consumption in humans

**DOI:** 10.1038/s41598-024-64556-w

**Published:** 2024-06-13

**Authors:** Leyre Notario Barandiaran, Vivien F. Taylor, Margaret R. Karagas

**Affiliations:** 1 Department of Epidemiology, Geisel School of Medicine, Dartmouth College, 1 Rope Ferry Road, Hanover, NH 03755-1404 USA; 2 Department of Earth Science, Dartmouth College, 6105 Sherman Fairchild Hall, Hanover, NH 03755 USA

**Keywords:** Seaweeds, Arsenic, Iodine, Biomarker exposure, Feeding study, Environmental impact, Metals, Epidemiology

## Abstract

Seaweed consumption has gained popularity due to its nutritional value and potential health benefits. However, concerns regarding the bioaccumulation of several trace elements highlight the need for comprehensive studies on exposure associated with seaweed consumption. To address this gap in knowledge, we carried out a feeding intervention study of three common edible seaweeds (Nori, Kombu, and Wakame) in 11 volunteers, aiming to elucidate the extent of both beneficial and harmful trace element exposure through seaweed consumption in humans. Concentrations of total arsenic, cobalt, copper, cadmium, iodine, molybdenum, selenium, and zinc were measured in urine samples before and following seaweed consumption. Elements concentrations were also measured in the seaweeds provided for the study. Descriptive analysis for each element were conducted and we used quantile g-computation approach to assess the association between the 8-element mixture and seaweed consumption. Differences in urine element concentrations and seaweed consumption were analyzed using generalized estimating equations (GEE). Urinary concentrations of iodine and total arsenic increased after seaweed consumption. When we analyze the 8-element mixture, the largest weight was observed for iodine after Kombu consumption while for total arsenic was observed after Wakame consumption. Similar results were observed when we compared the mean differences between the elements before and after seaweed consumption through the GEE. Seaweed consumption relates with increased urinary iodine and total arsenic concentrations, particularly after Kombu and Wakame consumption.

## Introduction

Seaweed refers to macroalgae, which are macroscopic and multicellular plant-like aquatic organisms. Unlike terrestrial plants, seaweeds exhibit remarkably rapid growth rates, and the lack of a distinct root, stem, and other anatomical structures typical of land-based vegetation^1^. These organisms fall into three phyla groups: Chlorophyta (green algae), Phaeophyta (brown algae), and Rhodophyta (red algae)^2^, which gives them the different pigments as well as biochemical and cellular characteristics^3^.

Seaweeds play an integral role in traditional Asian cuisine, particularly in China, Japan, and Korea. In the United States (US), the consumption of seaweed has been steadily increasing by approximately 7% annually, primarily due to its enhanced commercial availability^4^. The rise in seaweed’s popularity may in part be due to the marketing of seaweed products as makes “superfoods” due to their minerals, vitamins, proteins, fiber, and low-fat content^5,6^. Seaweeds contain soluble fiber, such as carrageenan and alginic, compounds associated with beneficial effects on digestive health^7,8^. In terms of mineral content, seaweeds boast elevated levels of calcium, zinc, potassium, copper, and iron, surpassing even meat and spinach in iron and copper content. Iodine is also prominent in seaweed; this essential micronutrient is required for the synthesis of thyroid hormones and the prevention of iodine deficiency related conditions^9,10^. Seaweeds also represent one of the few vegetable sources of vitamin B_12_, highlighting their potential value for individuals adhering to plant-based diets^11^. Indeed, there is some, however, limited evidence that seaweed consumption may offer protective benefits against cardiovascular diseases, diabetes mellitus, and hypertension^6,12^.

While seaweeds are a valuable nutritional source of iodine, certain types of brown seaweeds such as *Laminaria* and *Saccharina genus* (Kombu), contain excessive iodine levels, reaching values as high as 2000 and 6000 mg/kg dry weight^13,14^, and such concentrations may be detrimental to health^15,16^. Therefore, the European Scientific Committee on Food established an upper limit intake for iodine of 600 μg for adults^17^. Seaweeds likewise bioaccumulate non-essential elements such as arsenic and cadmium, which have negative health effects even at low concentrations^18–20^. In prior studies, brown seaweeds had higher concentrations of total arsenic compared to red and green seaweeds^21–23^. Most seaweeds contain organic forms of arsenic (arsenosugars), considered less toxic or for which the toxicity is not known. However, the brown seaweed Hijiki (*Sargassum fusiforme*) contains concerning concentrations of inorganic arsenic, and advisories about its consumption have been issued by countries like Canada, the United Kingdom, Australia-New Zealand, and China^24^.

Despite the nutritional benefits of seaweed and the potential for toxic element exposures, few studies have related seaweed intake to biomarkers of trace elements in humans. Our study aimed to determine human exposure to both essential and non-essential elements, and which elements were most strongly related to consumption, through a three-week intervention study of common edible seaweeds marketed in the US.

## Results

We enlisted 11 adult volunteers, of which 7 were women and 4 were men, with ages ranging from 24 to 61 years and body mass index from 18.4 to 28.5 kg/m2.

The concentrations of elements analyzed in the three different seaweeds consumed in this feeding study are presented in Table 1. For total arsenic, cadmium, zinc and cobalt, the highest concentrations were found in Wakame with concentrations of 45.91, 1.74, 88.55 and 0.10 μg/g, respectively. For iodine, a high iodine concentration of 3522 μg/g was observed in Kombu, whereas much lower iodine concentrations of 5.0 μg/g and 27 μg/g were determined for Nori and Wakame, respectively. For copper, the highest concentration was observed in the seaweed Nori (3.36 μg/g).

**Table 1:** Seaweed element concentrations and mean estimated recovery of elements in urine relative to total elements consumption.

Element	Nori		Kombu		Wakame	
	Concentration	Recovery	Concentration	Recovery	Concentration	Recovery
	(µg/g)	(Mean ± sd) (%)	(µg/g)	(Mean ± sd) (%)	(µg/g)	(Mean ± sd) (%)
As	17.80	31.11 ± 31.80	45.46	11.10 ± 11.20	45.91	21.27 ± 19.20
Cd	0.47	6.41 ± 7.35	0.49	8.00 ± 12.02	1.74	1.52 ± 1.51
Co	0.05	91.56 ± 117.42	0.03	134.73 ± 188.81	0.10	39.00 ± 39.27
Cu	3.36	30.48 ± 44.25	0.95	80.09 ± 65.42	2.69	28.27 ± 17.53
I	5.00	5890.86 ± 6720.05	3522.00	19.81 ± 18.24	27.00	2076.54 ± 1204.59
Zn	18.33	159.42 ± 303.89	14.43	143.43 ± 117.99	88.55	28.65 ± 22.30

Recovery was estimated multiplying elements concentrations (D1, D2, and, D3) by the volume in the 24 h urine sample and dividing by the amount of elements in the daily seaweed 10 g portion consumed.

At baseline we assessed the correlation matrix between urinary element concentrations (Figure S1). Before seaweed consumption the highest correlations were observed between cobalt and cadmium with a rho (r) = 0.61 (*p* value = 0.03) and cobalt and copper with a r = 0.65 (*p* value = 0.03) (Figure S1). The correlation between urinary element concentrations was also determined after seaweed consumption (Figure S2). The highest correlations were observed between zinc and selenium with a r = 0.67 (*p* value = 0.01), and copper and cobalt with a r = 0.66 (*p* value = 0.05). When we determine the correlations by type of seaweed consumed (Figure S2), we observe similar correlations in the 3 different types of seaweeds, being the highest correlations for Nori, with a r = 0.69 (*p* value = 0.03) between cobalt and copper and a r = 0.78 (*p* value < 0.01) between zinc and selenium. The highest correlation between total arsenic and iodine was observed in the seaweed Kombu, with a r = 0.50 (*p* value = 0.124).

**Figure 1:**
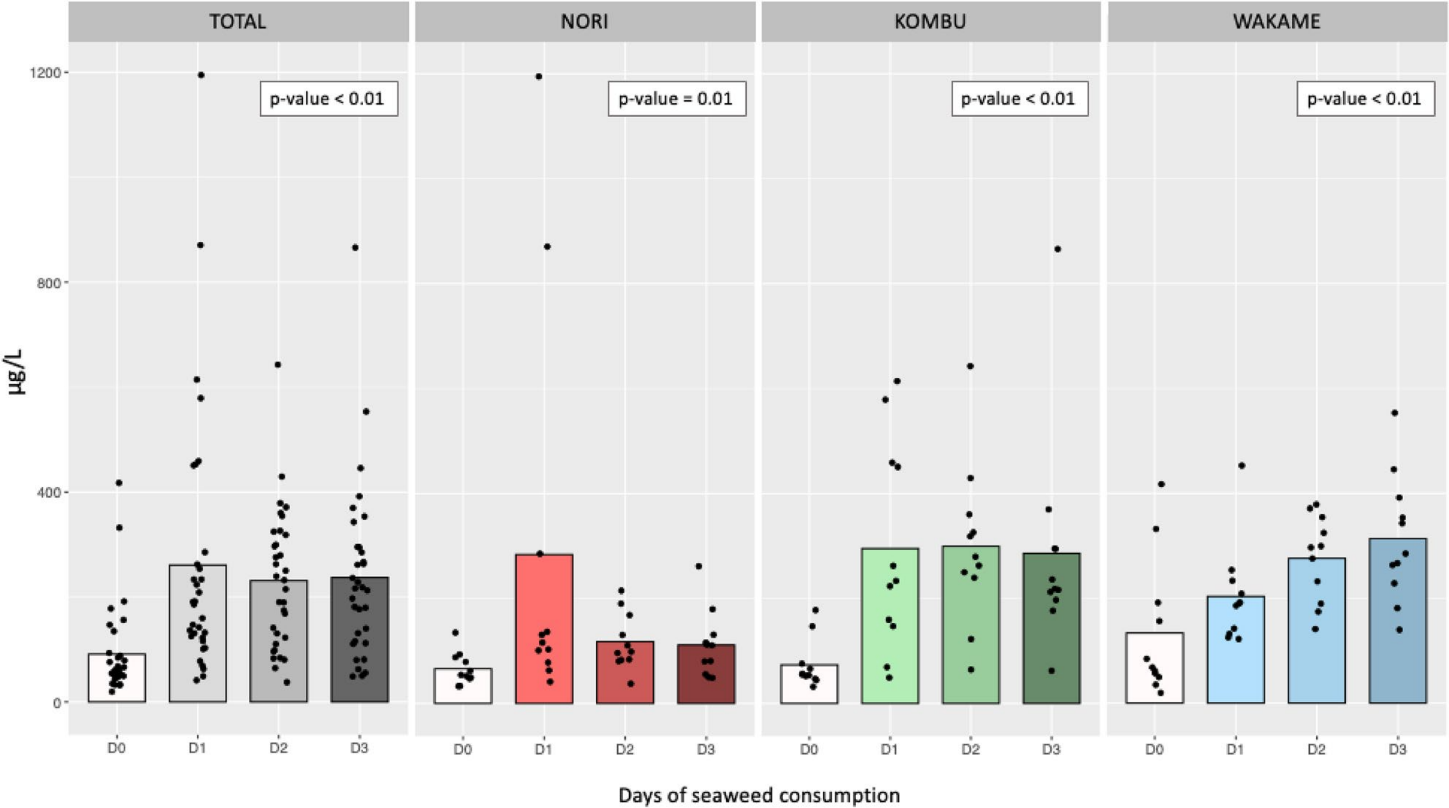
Mean iodine concentrations by day and type of seaweed consumed. Urine metal concentrations are normalized by specific gravity; D0 are urine metal concentrations at baseline (before seaweed intake); *p* value from urine metal concentrations mean difference between D0 and the average of D1, D2, and D3 assessed by generalized estimating equations.

In descriptive analysis, of all the elements analyzed, those for which significant increases in concentrations after seaweed consumption were observed were total arsenic and iodine (Fig. 1 and Table S1). Findings regarding arsenic have been published in greater details previously^22^, therefore were not the focus of our analysis except to understand the relative importance of arsenic and other elements following each of the seaweed interventions.

**Figure 2:**
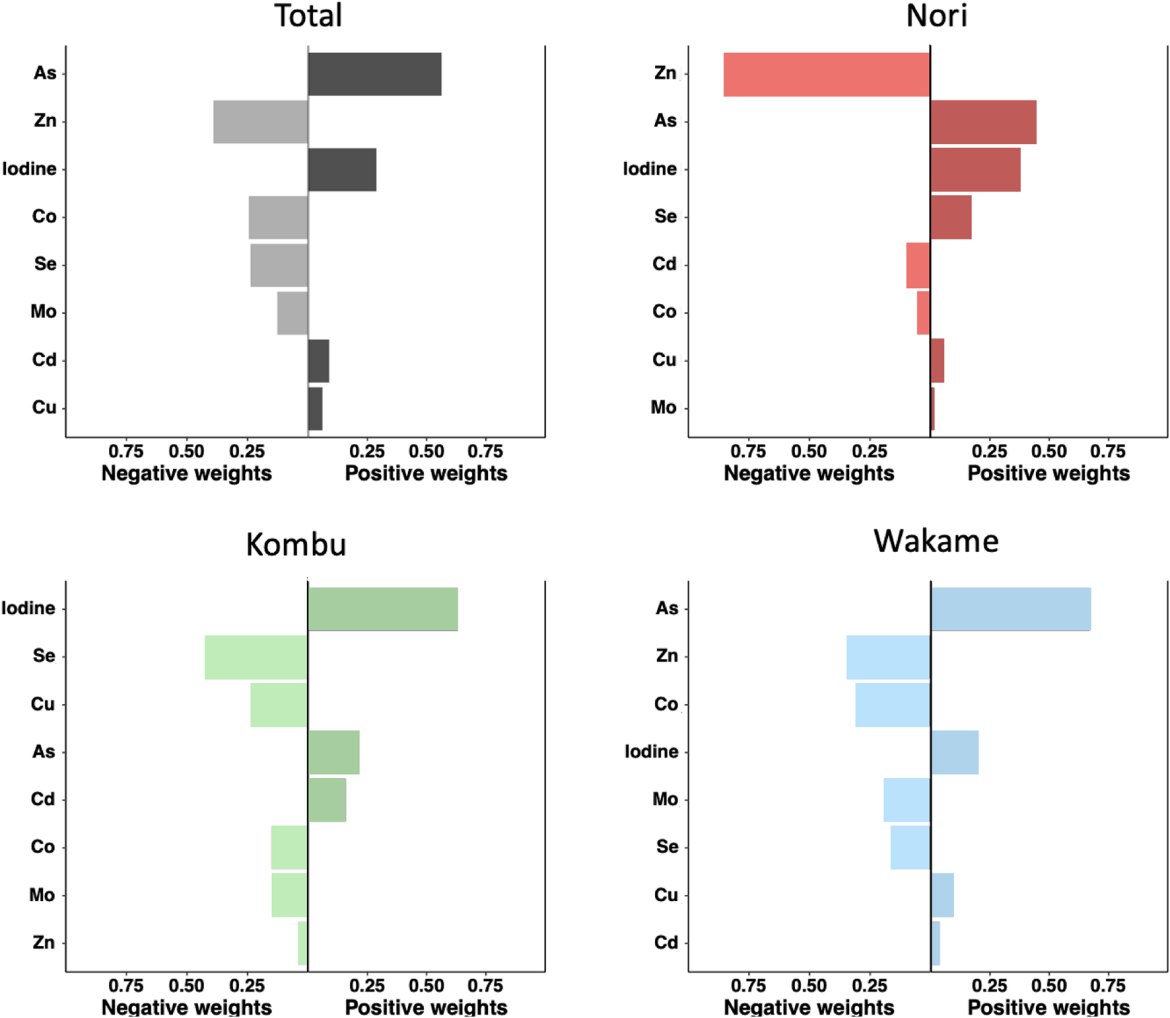
Quantile g-computation approach between metal mixtures and total and by type seaweed consumption (before vs. after).

At baseline (D0) the mean (sd) concentration of urinary iodine was 91.1 (85.2) μg/L which was increased to 237 (166) μg/L after three days of seaweed consumption (Fig. 1 and Table S2). When we examined urinary iodine concentrations by type of seaweed, we observed the highest concentrations after consumption of both brown seaweeds (Fig. 1 and Table S3). After the consumption of Kombu, urinary iodine concentrations increased from a mean (sd) of 73.4 (45.9) μg/L at D0 to 286 (207) μg/L at D3. Before Wakame consumption (D0), the mean (sd) urinary iodine concentration was 134 (131) μg/L and increased to 314 (120) μg/L after three days (D3). Urinary iodine concentrations also increased after consumption of Nori seaweed, although to a lesser extent, with a mean (sd) of 66.0 (30.5) μg/L at D0 and 111 (63.5) μg/L at D3 (Fig. 1 and Table S3). Figure 1 also displays the concentrations per day and type of seaweed across participants, showing a great individual variability.Figures are not in sequential order in the article. Please confirm if it is ok to proceed with the order provided.The order that the figures and tables are presented in the article is correct. Table 1 first, and figure 1 and figure 2.

We examined the element mixture in relation to seaweed consumption (before vs. after) using the quantile g-computation approach. Within the sum of 1 over all weights, total arsenic had a positive weight of 0.56 and iodine had a positive weight of 0.29 (Fig. 2). When we performed the mixture analysis by type of seaweed consumed, for Nori, total arsenic and iodine were the elements with the highest positive weights, with weights of 0.40 and 0.38, respectively. For Kombu, iodine stood out with a positive weight of 0.63. For Wakame, total arsenic showed a positive weight of 0.67 with a smaller positive weight of 0.20 for iodine (Fig. 2). Regarding negative weights, zinc was the element with the most negative weight after seaweed consumption with a weight of 0.37. The highest negative weight by type of seaweed for zinc was observed after Nori consumption with a weight of 0.83 (Fig. 2).

Using GEE analyses, we computed the percent differences in urinary element concentrations between D0 (before) and the average of D1, D2, and D3 (after) seaweed consumption (Fig. 1 and Table S4). For iodine, we observed a 194% increase in urinary concentrations after total seaweed consumption compared to iodine urinary concentrations before consumption. The percent increase in urinary iodine concentrations before vs. after consumption varied according to the type of seaweed consumed. Kombu had the highest percent change after consumption with an increase of 301%. For Nori and Wakame, increases of 118% and 186% was observed after consumption respectively (Fig. 1 and Table S4). For the rest of the elements analyzed, we did not observe appreciable differences between the concentrations before and after seaweed consumption.

Mean estimated elements recoveries by seaweeds were presented in Table 1. Iodine concentrations were highest in Kombu seaweed, however the recovery from Kombu seaweed was the lowest. The highest iodine recovery was observed for Nori seaweed, which had the lowest iodine concentrations when analyzed (Table 1). For the rest of the elements analyzed, we observed recoveries close to 100% or higher for cobalt and zinc after consumption of Nori and Kombu seaweed.

## Discussion

In this human experimental study, we examined eight elements before and during the three days feeding intervention with three different seaweeds. Of the elements analyzed, total urinary arsenic and iodine were both associated with seaweed consumption. When evaluating nutrient and toxic elements as a mixture, iodine had the highest weight after Kombu consumption and total arsenic had the highest weight after Wakame consumption. Total arsenic also showed the highest weight after Nori consumption, yet the weight was relatively lower than that observed for Wakame. Relatively high levels of iodine were found in Kombu seaweed, while Nori and Wakame seaweeds, showed considerably lower levels.

Our findings on urinary and seaweeds concentrations align with the previous literature on seaweed, with higher concentrations of iodine and total arsenic in the brown seaweeds, e.g., Kombu and Wakame, and lower concentrations in red seaweed e.g., Nori^25,26^. In studies of seaweed elements, Kombu is also the product with the highest iodine concentrations of the brown seaweeds tested^14,27^. In an Italian study, 14 seaweeds were analyzed, with findings of highest iodine concentrations for Kombu (7316 mg/kg dry)^28^. Similarly, in a study from Norway, 96 different seaweed products from different countries were analyzed; Kombu was the third seaweed with the highest iodine content (2.276 μg/g) after oarweed, and sugar kelp^14^. In a study of 30 different samples of Kombu from Asia and Europe, highest iodine concentrations were detected in the European Kombu (27.7 ± 5.4 mg/kg dw)^26^. In this study, we observed high concentrations of iodine in urine following the consumption of Kombu and Wakame. After consumption of Kombu, urinary iodine concentrations remained consistent throughout the days of intake, whereas with Wakame, an increase in concentrations was observed over the days of consumption. Conversely, for Nori seaweed, which is characterized by low iodine concentrations, we observed a high urinary iodine mean concentration on the first day of consumption due to the high concentrations in two participants. These high values may be due to iodine from other food sources. The overall median urinary iodine concentrations over the days remained similar, however. The concentrations of elements such as iodine can vary substantially with the processing or cooking type of the seaweed. Previous studies show how certain edible seaweeds when boiled reduce their total arsenic content by 43–50%^29,30^. For the seaweed Kombu, iodine concentrations can be reduced by up to 99% when the seaweed is boiled for 15 min^31^. Soaking seaweed has also been considered as a way of cooking that reduces total arsenic content by up to 60%^32^. Despite the reduction in iodine and other elements after processing, the amount of elements exposure through seaweed intake may still be of concern. Another concern related with seaweeds consumption is the labelling of the products. Several studies have shown incorrect labeling of seaweed products. In previous studies, about 12–16% of the seaweed products analyzed had incorrect labeling where the type of seaweed consumed was not declared or not include labelling of any sort^14,27^. Finally, the presence of different iodine species has been suggested to occur in seaweeds^33^, which may have different bio-availabilities. However, this study found iodide to be the primary iodine species in all three seaweeds (Figure S3), a finding similar to other studies^33^. While differences in iodine speciation are not evident between seaweeds, digestibility of the matrix of different seaweeds could affect element concentrations in urine.

Our results have potential health implications. Iodine is required for the synthesis of thyroid hormones and in turn proper metabolic function of multiple systems. However, both deficient and excess iodine can have adverse impacts. Excess iodine exposure through seaweed consumption has been associated with hyperthyroidism, thyroiditis, goiter, and kidney damage^34,35^. Children and pregnant people may be especially vulnerable to excessive iodine intake due to their smaller thyroid gland size and its possible adverse effects on fetal development^36^. Indeed, in a study of an Asian pregnant population, consumption of seaweed during pregnancy and lactation associated with iodine-induce hypothyroidism in the newborn^37^. These effects may be reversible, at least in adults evidenced by an experimental study of normal Japanese adults in which suppression of thyroid function with Kombu consumption declined when consumption of the seaweed was avoided^38^.

As mentioned, seaweed is a rich source of nutrients, such as vitamins, minerals, flavonoids, and dietary fiber^12^. Further, the production of seaweed requires fewer resources compared to seafood aquaculture, traditional plant-based agriculture, and livestock farming. Thus, does not raises fewer concerns about greenhouse gas emissions and pressure on natural ecosystems than other food production^39,40^.

Our experiment had several limitations. The washout period between seaweed consumption may not have been sufficient, leading to ”spillover effects”. For iodine, initial concentrations (D0) did increase from the first intervention, seaweed A, (66 μg/L) to seaweed C (133.8 μg/L). For the rest of the elements, baseline concentrations (D0) remain similar across the three intervention periods. In addition, in this study, the abstinence was only from foods associated with arsenic, such as rice and fish, but participants did not abstain from foods rich in iodine. Not controlling for participants' intake of iodine-rich foods may introduce errors in interpreting the study results, including difficulty discerning the source of iodine excretion. Another limitation of this study is that we only evaluated exposure to eight elements through seaweeds, and we know that seaweeds are important sources of vitamins, fiber, and other nutrients.

This study also possesses strengths. To our knowledge, this is among the first studies assessing multi-element exposure through seaweed consumption in humans using a biomarker of exposure. As far as we know, only one study analyzed the association between Kombu consumption and urinary iodine concentrations in humans^38^. Moreover, we examined element mixtures associated with seaweed consumption, as a more realistic method of exposure assessment. It is also important to note that we observed high inter-individual variability even for a given type of seaweed that likely reflects differing levels of absorption and excretion among individuals and can play a major role in individual exposure to both beneficial and detrimental trace elements. Therefore, this large differences on exposure found in the different participants should be further explored in future studies.

In conclusion, our study highlights the association between dietary seaweed intake and elevated urinary concentrations of total arsenic and iodine in humans after a period of 3 day seaweed consumption that was especially evident for Kombu and Wakame. Importantly, the scarcity of research assessing human exposure to both essential, and non-essential elements through seaweed consumption underscores the urgency for further investigation. Future studies should explore the mixture effect of different types of seaweed consumption, evaluate additional dietary sources of iodine, conduct longitudinal assessments to elucidate long-term effects of seaweed consumption, and investigate the influence of processing methods on exposure levels. Addressing these gaps will not only enhance our understanding of the health risks and benefits associated with seaweed consumption but also provide valuable insights for establishing public health policies and dietary recommendations.

## Methods

### Study population and design

**Figure 3:**
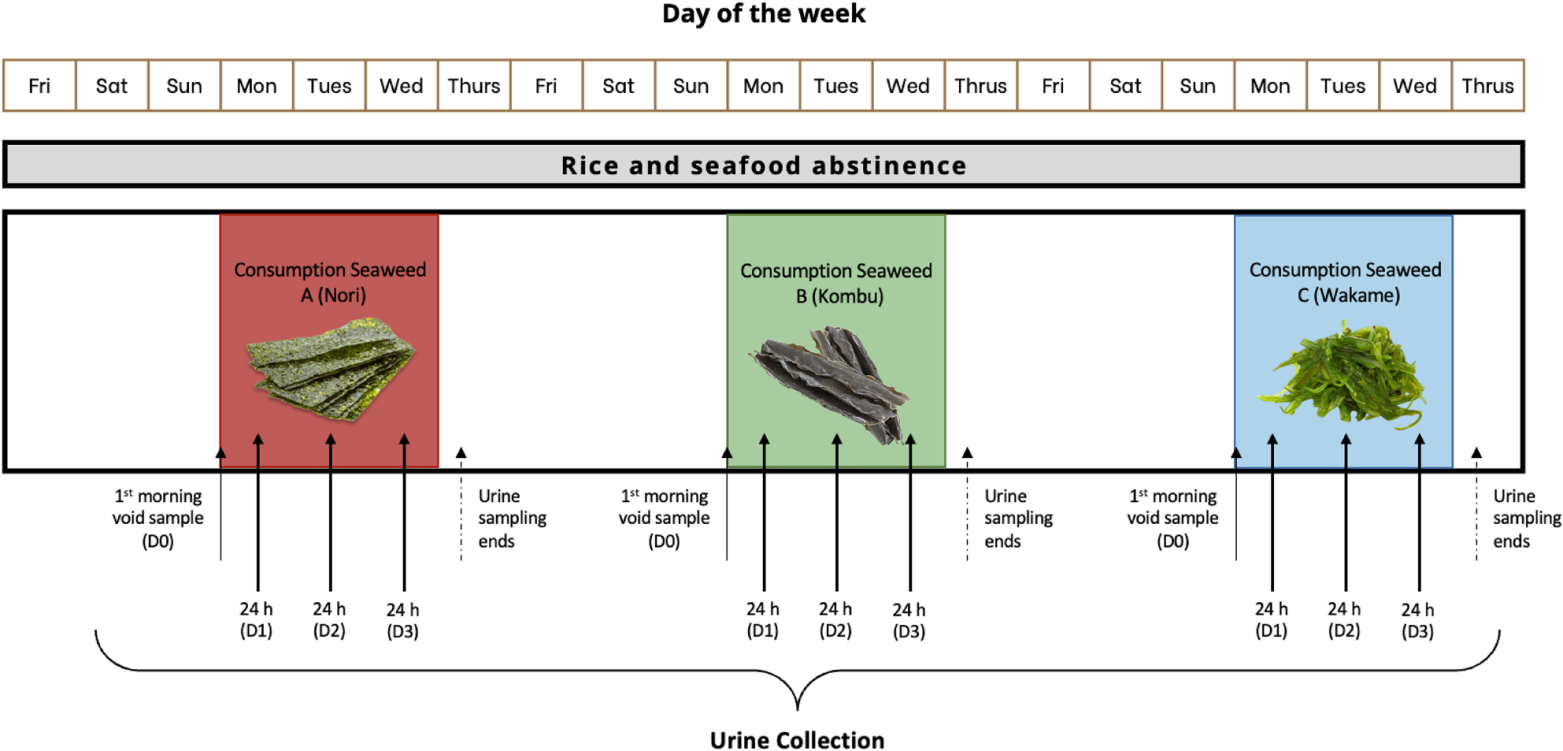
Experimental feeding study design.

An experimental feeding study was conducted with eleven volunteers using three of the most consumed seaweeds worldwide^5,22^. Nori (Porphyra) is an edible seaweed that belongs to the group of red algae and is usually consumed dried and is cultivated mainly in Japan^41^. Kombu (Laminaria) is a thick flat seaweed belonging to the brown algae group and is traditionally used to prepare Japanese soup stock^42^. Wakame (Undaria) also belongs to the brown algae group. Its flavor is subtly sweet and is usually consumed in soups or salads^43^. Each participant received pre-weighed portions of 10 g of each type of seaweed and was provided with written instructions and necessary materials to carry out the experiment. The experimental feeding study was conducted in three different blocks (Fig. 3), each block involving the consumption of a different type of seaweed. In the first block, Nori (Seaweed A) was consumed raw. In the second block, Kombu (Seaweed B) was consumed soaked in 125 mL water for 1 min. Finally, in the third block, Wakame (Seaweed C) was consumed soaked in 125 mL water for 10 min. Before starting each of the three blocks of the feeding study, first-morning urine samples were collected (D0). The element concentrations at D0 were used to determine the concentrations at baseline (before seaweed consumption). During the following three days (D1, D2, and D3) in which the designated seaweed for each block was consumed, 24h urine samples were collected. All participants refrained from fish and rice consumption from three days before the start of the study until the end. In addition, a 4-day washout period was set between blocks.

The participants were informed of the study's objectives and provided written informed consent prior to their participation. The experimental protocol was approved and conducted in accordance with the guidelines and regulations of the Dartmouth College Committee for the Protection of Human Subjects (CPHS#: STUDY00028666). Dartmouth College CPHS approved the protocol, which stated the serving sizes and concentrations of elements in each of the seaweeds, as well as the participant recruitment strategy and the participant consent forms.

### Trace element concentrations in seaweed

Concentrations of trace elements in seaweeds were determined by two methods. For trace metal(loid) concentrations, pulverized seaweed samples were digested in strong acid under high pressure in a microwave. Seaweed samples were weighted into 10 mL Teflon vessels and 5 mL concentrated HNO_3_ was added to each vessel. Samples were heated to 190 °C for 10 min by high pressure microwave digestion, then transferred to pre-weighed 60 mL vials, and diluted to 50 mL with ultrapure water, and weighed. Seaweed samples were diluted by mass another 20-fold with 1% HNO_3_ into 7 mL vials for analysis. Element concentrations of arsenic, cadmium, cobalt, copper, and zinc were determined by collision cell ICP-MS with a collision gas flowrate of 5 mL/min. Recoveries of each element in two standard reference materials are given in Table S1. For iodine determination, seaweeds were digested in basic solution, as iodine becomes volatile under acidic conditions. Aliquots (0.25 g) of seaweed were weighed into 50 mL pre-weighed vials, and 5 mL of 25% tetramethylammonium hydroxide (TMAOH) were added to each vial and heated to 90 °C for 30 min in a microwave. Samples were then diluted with ultrapure water to 50 mL, and analyzed by ICP-MS. Duplicate precision was 8 ± 6%, and spike recovery of an iodine standard was 109%.

### Urine elements concentrations

Urine samples were collected in acid-washed vials, 250 mL vials for D0 and 500 mL vials for D1, D2, and D3, which were compiled in 3 L containers for each 24 h period. After collection samples were kept cool and delivered to the laboratory each day. Urine subsamples (15 mL) for each day were obtained and frozen at − 20 °C to analyze the concentrations of different elements. Before analysis, samples were thawed and vortexed. Samples were analyzed for total arsenic, cadmium, cobalt, chromium, copper, iron, manganese, molybdenum, nickel, antimony, selenium, vanadium, zinc, and iodine by triple quad inductively coupled plasma-mass spectrometry (8800 ICP-QQQ; Agilent, Santa Clara, CA) operated with oxygen as a reaction gas (0.3 mL/min). Quality control was assessed using calibration check standards, which were analyzed every 10 samples throughout each run, and duplicate and standard spike samples were monitored every 20 samples. The laboratory also participates in the quarterly CTQ Toxicologie de Quebec proficiency testing for serum and urine, which provides additional quality assurance. Recoveries of urine element concentrations range from 91% for Zn to 99% for manganese and iodine. The limits of detection (LOD) for total arsenic, cadmium, cobalt, chromium, copper, iron, iodine, manganese, molybdenum, nickel, antimony, selenium, vanadium, and zinc were 0.03, 0.01, 0.01, 0.50, 0.24, 8.00, 0.50, 0.08, 0.03, 0.24, 0.02, 0.06, 0.08, and 1.60 μg/L, respectively. Element concentrations below the LOD were assigned a value of the LOD divided the square root of 2^44^. The elements chromium, iron, manganese, nickel, antimony, and vanadium had 100%, 99%, 98%, 98%, 100%, and 100% of urinary concentrations below the LOD respectively and were therefore excluded from the analyses.

### Statistical methods

Two spot samples were missing for baseline (D0) for seaweed B (V10 and V11), so the average baseline (D0) urine element concentrations for seaweed A and C were assigned for these samples for those participants.

Before analysis, urinary concentrations of all elements were normalized by specific gravity using ${E}_{sg}={E}_{0}\hspace{0.33em}x\hspace{0.33em}\frac{S{G}_{median}-1}{S{G}_{0}-1}$ where E_sg_ is the specific gravity-standardized exposure biomarker concentration, E_0_ is the observed exposure biomarker concentration, SG _median_ is the median of specific gravity value in the study population, and SG_0_ is the observed specific gravity value^45^. Spearman correlation matrices of each pair of elements were calculated excluding concentrations at baseline (D0). As preliminary analysis, we examine urinary element concentrations as a mixture using the quantile g-computation approach. This method estimates the joint effect of the element mixture on seaweeds consumption (before vs. after) when increasing all metals by a single quantile. It indicates the weighted contribution of each individual metal of the mixture to the global estimation and does not require the same effect direction of exposures in the mixture. In addition, the quantile g-computation approach yields unbiased estimates of overall mixture effects in small sample sizes with acceptable confidence interval (CI) coverage^46^. To carry out these analyses we use the “qgcomp.noboot” function from the “qgcomp” package.

Descriptive statistics (mean and standard deviation (SD)) were performed for total urine concentrations of each element by day of consumption and type of seaweed consumed. To test the difference between urine element concentrations before (D0) and after seaweed consumption (average of urine element concentrations at D1, D2, and D3) we used the semiparametric regression method: generalized estimating equations (GEE). The variable “Day of consumption” was categorized in two dichotomous categories “Before consumption” or “After consumption” and was included in the models as the independent variable with each urine element concentration included in the model as dependent variables. The percent change in urine element concentration by day of consumption (after vs. before) was calculated from the β from the GEE models using the formula: exp(β-1)*100. We calculated GEE for both the total seaweed consumed and for each type of seaweed consumed. For GEE analysis urine element concentrations were log-transformed.

Elements recoveries were calculated after seaweed consumption. Percentage of recovery of each element was calculated from urinary concentrations measured in the 24h samples and divided by the elements concentrations measured in the 10 g of the seaweed consumed. Mean recoveries were reported for each seaweed type.

All statistical analyses and graphics were performed with R version 4.1.2^47^.

### Supplementary Information

Supplementary Information.

## Data Availability

The dataset generated and analysed during the current study are available from the corresponding author on reasonable request.
