# Supplementary material for: Exposure to iodine, essential and non-essential trace element through seaweed consumption in humans
Source: Sci Rep. 2024 Jun 13;14:13698. doi: 10.1038/s41598-024-64556-w (PMC11176391; doi:10.1038/s41598-024-64556-w)
Supplement: Supplementary file 1 — Supplementary Information. [file 41598_2024_64556_MOESM1_ESM.pdf]

Exposure to iodine, essential and non-essential trace element through seaweed consumption in humans.

Leyre Notario Barandiaran[1*], Vivien F. Taylor[2], and Margaret R. Karagas[1]

Figure S1. Spearman matrices correlation between metals concentrations at baseline (D0) for both total and each seaweed consume.

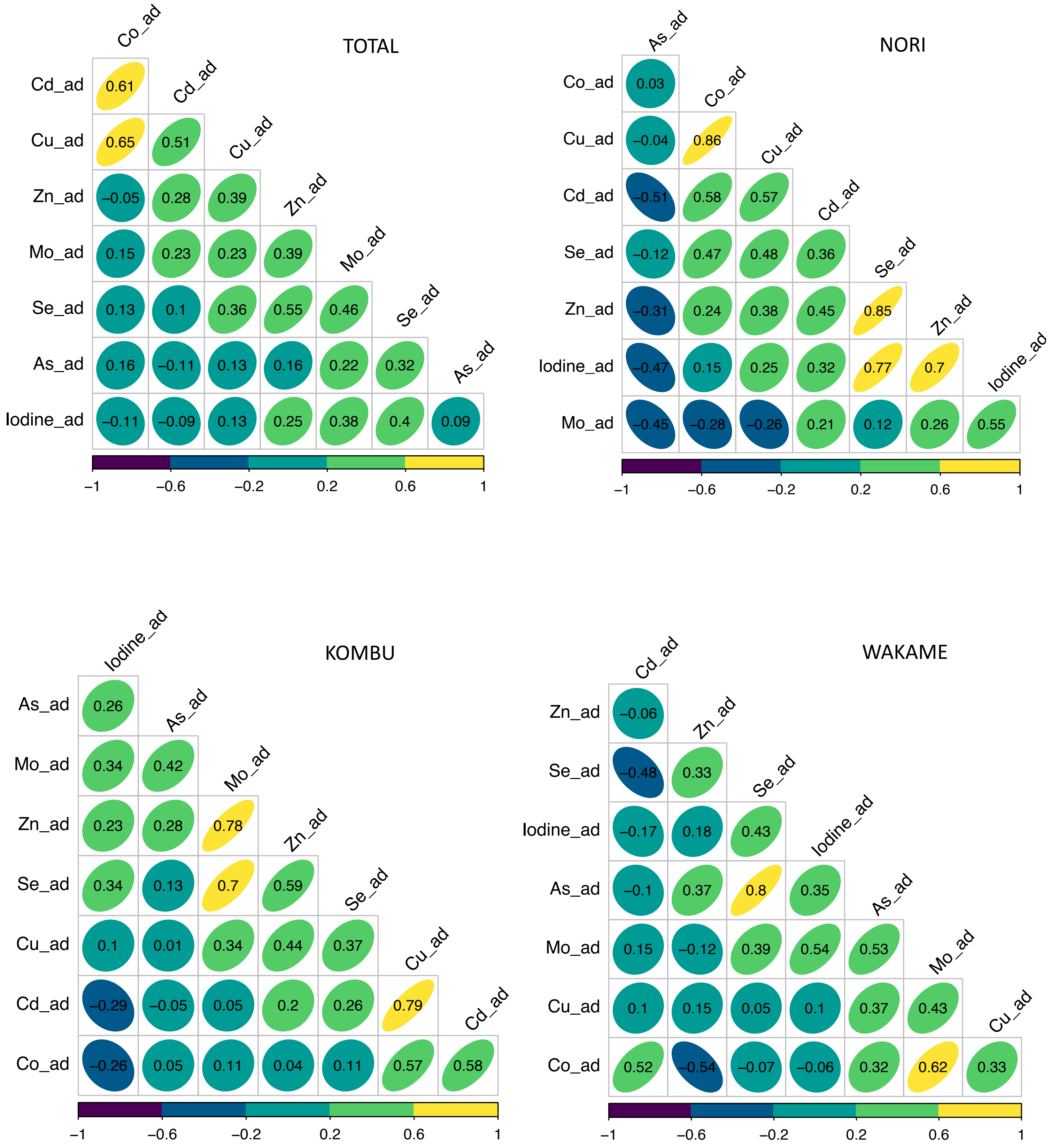

Figure S2. Spearman matrices correlation between metals concentrations after seaweed consumption (excluding concentrations at D0) for both total and each seaweed consume.

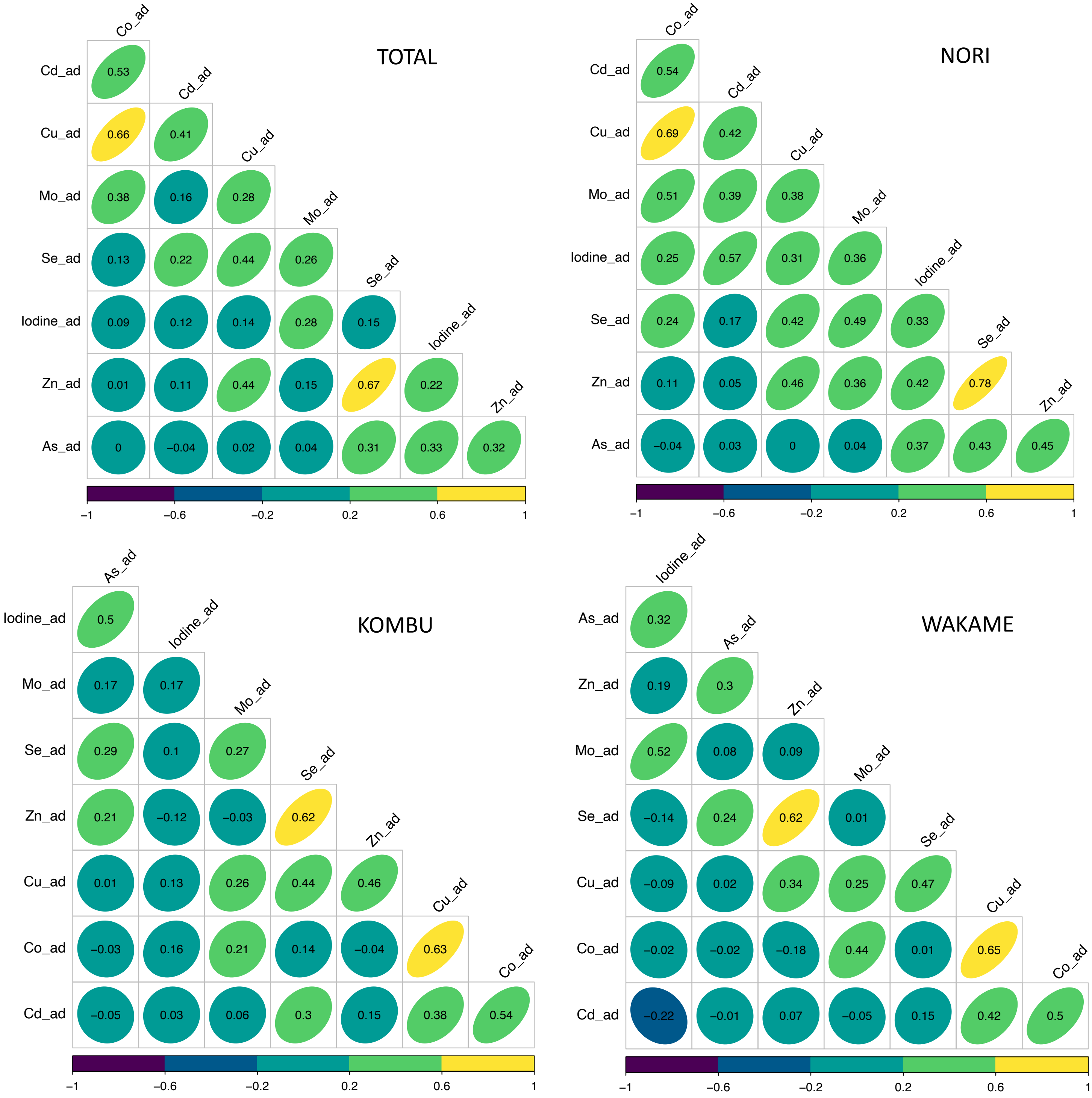

Figure S3. Chromatograms of a multi-species iodine standard (I std) containing iodate, iodide, 3,5-diiodo-tyrosine (DIT), and 3-iodo-tyrosine (MIT), overlaid with extracts of Nori, Kombu and Wakame. Samples were analyzed by size exclusion chromatography coupled with ICP-MS, using a Superdex 75 column, a mobile phase of 20mmol ammonium acetate (pH 7.4) at a flowrate of 0.7 ml/min, and an injection volume of 20 µl.

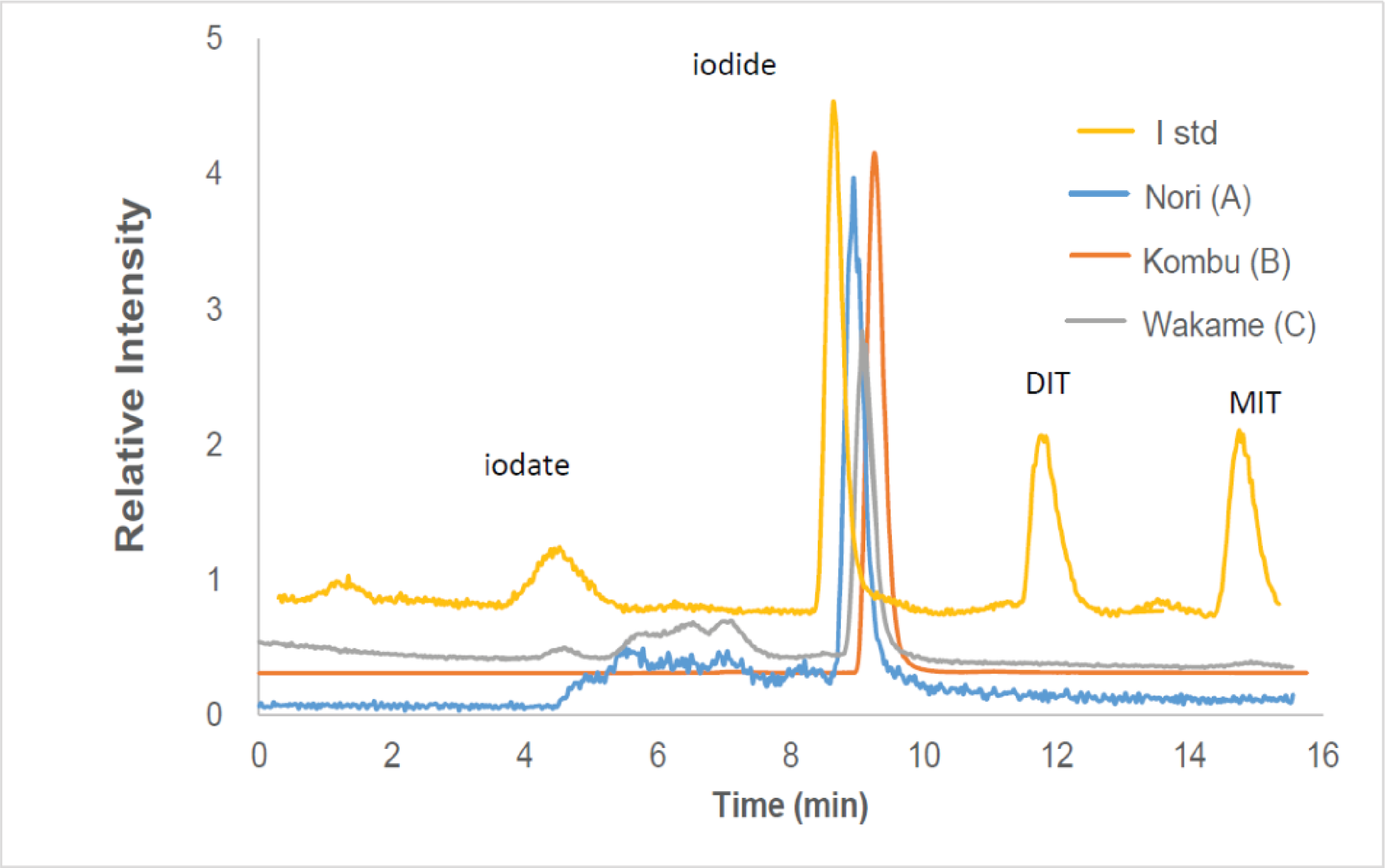

Table S1. Certified values (recommended values in italics) of trace element concentrations (in mg kg^{-1}) in Certified Reference Materials DOLT-4 (National Research Council of Canada) and Oyster 1566b (NIST), along with analytical recoveries (%).

	**As**	**Cr**	**Co**	**Ni**	**Cu**	**Zn**	**Se**	**Se**	**Mo**	**Cd**
DOLT 4 known values (mg kg^{-1})	9.66	*1.4*	0.25	0.97	31.2	116	8.3	8.3	*1*	24.3
DOLT 4 recovery (%)	92%	*86%*	137%	120%	99%	98%	93%	101%	*120%*	90%
NIST OYSTER 1566b known values (mg kg^{-1})	7.65	N/A	0.371	1.04	71.6	1424	2.06	2.06	N/A	2.48
NIST OYSTER 1566b recovery (%)	103%	N/A	130%	109%	99%	102%	102%	115%	N/A	107%

Table S2. Descriptive analysis of urinary metal concentrations according to the day of seaweed intake.

Urinary metals concentrations (µg/L)*	D0 (mean±sd)	D1 (mean±sd)	D2 (mean±sd)	D3 (mean±sd)	p-value
Co	0.018±0.020	0.020±0.022	0.018±0.021	0.018±0.019	0.970
Cu	1.889±6.019	0.563±0.825	0.326±0.160	0.322±0.164	0.410
Zn	20.1±17.0	21.1±38.0	14.3±16.5	13.0±14.5	0.990
**As**	**0.438±0.245**	**1.896 ±1.411**	**3.545±2.616**	**4.221±3.656**	**0.035**
Se	2.492±1.382	3.396±7.251	2.183±1.390	1.962±0.919	0.950
Mo	2.78±2.31	2.31±2.01	2.09±1.30	1.81±1.42	0.860
Cd	0.019±0.013	0.018±0.020	0.011±0.010	0.013±0.010	0.320
**Iodine**	**91.1±85.2**	**260.7±250.1**	**231.1±130.5**	**237.0±165.8**	**0.035**

n=11; * Concentrations in urine samples, normalized to specific gravity; p-value from ANOVA paired test

Table S3. Descriptive analysis of urinary metal concentrations according to the day of seaweed intake by type of seaweed

Urinary metals concentrations (μg/L)	D0 (mean±sd)	D1 (mean±sd)	D2 (mean±sd)	D3 (mean±sd)
**NORI**				
Co	0.023±0.030	0.028±0.0288	0.0180±0.022	0.019±0.026
Cu	4.928±10.031	0.929±1.356	0.327±0.141	0.278±0.136
Zn	24.9±20.8	34.4±61.9	14.2±18.1	10.6±11.1
As	0.507± 0.240	2.399±1.730	2.810±2.171	2.803±1.783
Se	2.575±0.975	5.935±12.486	2.106±1.104	1.816±0.715
Mo	2.813 ±1.357	3.329±3.126	1.795±0.929	1.420±0.783
Cd	0.023±0.014	0.024±0.026	0.007±0.005	0.013 0.007
Iodine	66.0±30.5	283.6±382.5	117.6±53.3	111.4±63.5
**KOMBU**				
Co	0.016±0.014	0.013±0.014	0.020±0.023	0.016±0.016
Cu	0.379±0.185	0.310±0.152	0.335±0.175	0.309±0.147
Zn	18.6±16.3	13.1±15.5	12.8±16.6	11.4±13.6
As	0.361±0.224	1.515±1.323	2.591±1.810	2.591±1.882
Se	2.61±1.26	2.13±0.91	2.22±1.39	1.88±1.21
Mo	3.228±3.466	1.830±0.601	2.613±1.465	1.851±1.600
Cd	0.017±0.014	0.014±0.012	0.015±0.013	0.0143±0.010
Iodine	73.4±45.9	295.1±198.8	299.5±152.9	285.9±207.5
**WAKAME**				
Co	0.0147±0.012	0.018±0.018	0.016±0.019	0.018±0.016
Cu	0.359±0.169	0.449±0.289	0.316±0.177	0.380±0.199
Zn	16.9±13.7	15.6±16.1	15.7±16.2	16.9±18.5
As	0.445±0.269	1.775±1.083	5.235±3.030	7.269±4.550
Se	2.291±1.875	2.124±0.941	2.221±1.737	2.195±0.798
Mo	2.29±1.67	1.76±1.00	1.86±1.39	2.16±1.73
Cd	0.016±0.012	0.0160±0.021	0.012±0.010	0.013±0.014
Iodine	133.8±131.0	203.5±93.8	276.2±81.8	313.7±120.0

n=11; * Concentrations in urine samples, normalized to specific gravity

Table S4. Urine elements concentrations mean difference between D0 and the average of D1, D2, and D3 assessed by generalized estimating equations (GEE).

		**TOTAL**		**NORI**		**KOMBU**		**WAKAME**	
	D0	Average D1, D2, D3		Average D1, D2, D3		Average D1, D2, D3		Average D1, D2, D3	
Urine metal concentrations (μg/L)		% change (95%CI)	p-value	% change (95%CI)	p-value	% change (95%CI)	p-value	% change (95% CI)	p-value
Cobalt	Ref.	10.5 (-63; 99)	0.750	6.2 (-90; 116)	0.860	-3.1(-103; 90)	0.920	17.4 (-58; 120)	0.610
Copper	Ref.	-32.2 (-144; 38)	0.364	-103 (-511; 46)	0.200	-17.4 (-84; 32)	0.470	8.3 (-43; 68)	0.730
Zinc	Ref.	-28.4 (-164; 58)	0.490	-78.6 (-290; 20)	0.140	-58.4 (-278; 49)	0.300	13.9 (-93; 153)	0.740
**Arsenic**	**Ref.**	**582 (306; 1047)**	**<0.001**	**353 (166; 669)**	**<0.001**	**421 (177; 878)**	**<0.001**	**1070 (493; 2187)**	**<0.001**
Selenium	Ref.	-6.2 (-52; 36)	0.760	-8.3 (-80; 52)	0.730	-23.4 (-97; 28)	0.360	6.2 (-42; 60)	0.790
Molybdenum	Ref.	-16.2 (-86; 38)	0.530	-27.1 (-94; 20)	0.260	-27.1 (-112; 28)	0.340	1.0 (-159; 161)	0.990
Cadmium	Ref.	-22.1 (-114; 43)	0.470	-47.7 (-161; 19)	0.180	-22.1 (-120; 48)	0.510	-2.0 (-125; 116)	0.960
**Iodine**	**Ref.**	**194 (123; 282)**	**<0.001**	**118 (35; 253)**	**0.001**	**301 (156; 530)**	**<0.001**	**186 (65; 400)**	**<0.001**

D0, urine metal concentrations at baseline (before seaweed consumption); CI, confidence intervals
